# Epidemiological studies of burn patients in a burn center in Ghana: any clues for prevention?

**DOI:** 10.1186/s41038-016-0041-0

**Published:** 2016-07-11

**Authors:** P. Agbenorku, K. Aboah, J. Akpaloo, R. Amankwa, B. Farhat, E. Turkson, P. E. Hoyte-Williams, E. E. Klutsey, J. Yorke

**Affiliations:** 1 Reconstructive Plastic Surgery & Burns Unit, Department of Surgery, School of Medical Sciences, Kwame Nkrumah University of Science & Technology, Kumasi, Ghana; 2 Department of Surgery, School of Medical Sciences, Kwame Nkrumah University of Science & Technology, Kumasi, Ghana; 3 Department of Microbiology, School of Medical Sciences, Kwame Nkrumah University of Science & Technology, Kumasi, Ghana; 4 Reconstructive Plastic Surgery & Burns Unit, Department of Surgery, Komfo Anokye Teaching Hospital, Kumasi, Ghana; 5 Department of Nursing, University of Health & Allied Sciences, Ho, Ghana

**Keywords:** Burns, % TBSA, Scalds, Domestic setting, Outcome, Prevention

## Abstract

**Background:**

Burn injuries are a serious problem worldwide, with most occurrences in low- and middle-income countries. Depending on the extent of injury, burn victims are faced with the challenges of fitting into society due to complications such as extensive scarring and contractures. The current study seeks to determine whether epidemiological studies of burn patients can provide guidelines to enhance burn prevention among the Ghanaian population.

**Methods:**

Data from the Burns Registry of the Burns Intensive Care Unit (BICU) of Komfo Anokye Teaching Hospital (KATH) was obtained. Data on sex, age, aetiology, % total body surface area (TBSA), and admission outcomes from May 1, 2009, to April 30, 2013, were retrieved for a total of 487 patients during this period.

**Results:**

Data on burn admissions comprising 263 (54.0 %) males and 224 (46.0 %) females were obtained from the Burns Registry. Children 0–10 years were the most affected age group. The yearly mean % TBSA ranged from 24.74 % to 35.07 %. The majority of burns was caused by scalding. Mortality rates ranged from 8.4 % to 32.0 % during the period under review.

**Conclusions:**

The study shows that children of 10 years old and below are the most affected group; this may be due to inattention to these children by parents/caretakers. Safety and safe working environments should be provided at home and workplaces, and promotion of education on burn prevention should be intensified.

## Background

Burn injuries, a public health issue of high economic importance, continue to be a major problem both in developed and developing countries, affecting mostly children and the elderly [1–4]. Burns can occur by any of the following: flames, electricity, chemicals, hot liquids, or contact with any hot object. Severe scarring and contracture, as well as death, are complications from burn injuries. Therefore, it is critical that efforts be placed at preventing burns or having systems in place to manage burns when they occur. Burns account for 265,000 deaths annually, with approximately 95 % occurring in low- and middle-income countries (LMICs) [3]. Burn prevention and management involve a multi-centered approach targeted at helping the individual develop his or her self-esteem [5]. Severe scarring and contractures following burn injuries can greatly affect victims, as they may feel a loss of self; victims are often dependent on family members and friends for care, which leads to increased economic burdens. Burns are associated with increased hospitalization, scarring, and disfigurement [6]. The current study seeks to determine if epidemiological studies of burn victims can provide guidelines to enhance burn prevention among the Ghanaian population.

## Methods

### Study setting

Komfo Anokye Teaching Hospital (KATH), located in the middle belt of the country in the city of Kumasi, is the second largest hospital in Ghana. KATH was initially a 1000-bed unit but later expanded to 1500 to cater to the increasing number of patients. As a tertiary health facility, it serves as a referral center for people in the Ashanti, Brong Ahafo, and Northern and Upper Regions. It is affiliated to the School of Medical Sciences, Kwame Nkrumah University of Science and Technology. The Accident and Emergency (A&E) Center, established in 2009, is an ultra-modern facility with a 160-bed capacity and houses the six-bed Burns Intensive Care Unit (BICU) of the Reconstructive Plastic Surgery and Burns Unit (RPSBU) in addition to other departments.

### Data collection

A computerized database at the KATH BICU was used to retrieve data on demographics, aetiology, % total body surface area (TBSA), length of hospital admission, and outcome from May 1, 2009 to April 30, 2013.

### Data analysis

The data were analyzed using Statistical Package for Social Sciences (SPSS) version 16.0. Chi square test was performed, with *P* < 0.05 denoting significance.

### Ethical clearance

Ethical clearance for this study was obtained from the KNUST School of Medical Sciences/KATH Committee on Human Research, Publication and Ethics, Kumasi.

## Results

### Demographic features of patients

**Fig. 1:**
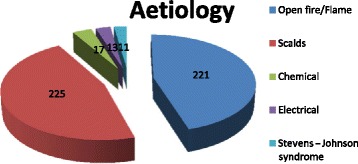
Aetiology of burns

**Fig. 2:**
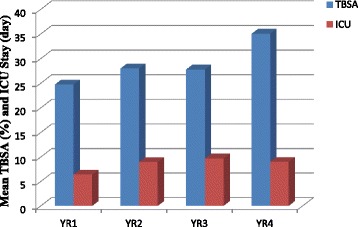
Yearly mean % total body surface area (TBSA) and intensive care unit (ICU) stay (day)

**Fig. 3:**
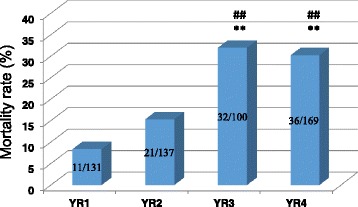
Yearly review of mortality rates. The first number is the deaths and the second is the admissions in each bar. The *asterisk* (*) indicates the results of the comparisons with *YR1*, and the *pound sign* (#) indicates the results of the comparison with *YR2*. A *P* value of 0.05 was considered significant

A total of 487 patient records from May 1, 2009 to April 30, 2013 were reviewed. Table 1 shows patient demographics, including sex distribution and age distribution. Table 2 shows the Haddon matrix. Figure 1 shows the aetiology of burns during the period under review; scalds were the most common cause (*n* = 225, 46.2 %), followed by open fire (*n* = 221, 45.4 %), chemical (*n* = 17, 3.5 %), electricity (*n* = 13, 2.7 %), and Stevens-Johnson syndrome (*n* = 11, 2.3 %). Figure 2 shows the yearly mean percent TBSA and ICU admission length. The mean % TBSA for the period under review was 28.79 %. Based on the yearly analysis, year 4 recorded the highest mean % TBSA (35.07 %, with a mean ICU stay of 9.02 days) followed by year 2 (27.98 %, with a mean ICU stay of 8.99 days), year 3 (27.76 %, with a mean ICU stay of 9.7 days), and year 1 (24.74 %, with a mean ICU stay of 6.39 days). Figure 3 shows a histogram and test results of mortality rate in different years. The mean mortality rate was 20.5 % for the period under review. A yearly review of mortality rate showed an upward trend from year 1 to year 4.

**Table 1:** Demography of patients

Item	Number of cases (%) Total = 487
Sex distribution	
Male	263 (54.0)
Female	224 (46.0)
Age distribution	
0–10	237 (48.7)
11–20	49 (10.1)
21–30	94 (19.3)
31–40	53 (10.9)
41–50	30 (6.2)
51–60	18 (3.7)
61–70	4 (0.8)
71–80	1 (0.2)
81–90	1 (0.2)

**Table 2:** Haddon’s matrix

	Host	Agent	Physical environment	Social environment
Pre-event	Keep children from source of fire	Keep children from source of fire	Provision of fire alarm systems	Close monitoring of children
	Ensure adherence to safety practices	Containers with hazardous chemicals should be stored at designated areas	Ensure standard operating procedures	Safe working environment
		Chemicals should be kept at clearly defined areas		Advocacy on burns prevention
Event	Teach persons to conduct themselves in ways to prevent excessive burns, e.g., stop, drop, and roll policy	Flooding the burns area with a lot of water	Flooding with lot of water	Swift response of the rescue team
Post-event	Get the victim farther from the injury site	Turn off electrical supply	Provide fire alarms	Rehabilitation for victims
	Call for help	Help quench fire	Swift response of the rescue team	Care and support
				Financial support for victims

## Discussion

The current study reveals a predominance of burns in males over females. Similar findings have been reported in various studies. This may be due to the adventurous nature of males and their greater desire to be active compared to females [7, 8]. A study by Othman and Kendrick [9] also showed a greater number of burns in males compared to females [9]. The current study revealed 0–10 years as the group most prone to burn injury. A study by Outwater et al. [10] reported young age, especially 1–3 years, as a risk factor for burn-related morbidity. The high prevalence rate of children with burn injuries could be attributed to a lack of or inadequate supervision by mothers and caretakers [10]. The current study also revealed that most of the burns took place in the domestic setting, especially in the kitchen, where cooking occurs.

The most common cause of burn admission was scalding. For children, this could be the result of their highly active nature, in which they may unwittingly grasp at hot liquids due to their lower cognitive ability compared to adults [11, 12]. Balseven-Odabasi et al. [13] also reported scalding as the major aetiology in their study [13].

Burns are associated with hospitalization of the patient for management. The extent of burn injuries and prognosis may determine how long the patient will be hospitalized. From the current study, the highest mean length of stay was 9.7 days. An average length of hospital stay of 18 days was reported by Chien et al. [14] in a study on burn patients in Taiwan [14]. Akerlund et al. [15] also reported 3 days as a median length of hospital stay in Sweden [15].

The highest mean % TBSA for the current study was 35.07 %. TBSA has been reported as a risk factor for mortality [16]. TBSA is also an important indicator on how a patient is to be managed, especially in children and the elderly, and could possibly influence the management strategy of burn victims.

Most of the burns that occurred in the current study were unintentional except a single acid assault case that was caused intentionally. Assault-related burns caused by the use of acids and bases are a common occurrence in some settings around the globe [17]. Psychologically, these burn survivors grow up developing a negative self-image of themselves, are stigmatized, and sometimes may not even be employed or married [18].

Mortality has been reported as a complication of burn injury [19–22]. Olaitan and Jiburum [23] reported a burn mortality of 20 % out of 285 burn patients at a burn center in Nigeria in a retrospective study from January 1996 to December 2000 [23]. Ibran et al. [24] reported a mortality of 36.12 % in a 2-year prospective cross sectional study conducted in Karachi, Pakistan [24].

A burn injury, depending on the severity, may require the patient to undergo a series of surgical procedures, such as excision and grafting. In other circumstances, amputation may be suggested as a modality in the management of the victim. Surgical procedures are quite expensive, especially plastic surgery, reconstructive procedures, and cosmetic procedures [25–27].

Strict supervision of children is important for the prevention of burn injury. Public education on burn prevention and use of fire alarm systems should be encouraged.

Haddon’s theory on conceptualization, which is focused on prevention, was adopted [28]. At the workplace, standard operating guidelines and safety rules should be enforced to prevent burn occurrences [28].

## Conclusions

Burn injuries affect people of all ages, especially children below 10 years old. As such, these children require close supervision. Awareness of burn prevention and management should be promoted. Safe practices should be enforced at workplaces, including the presence of alarms and extinguishers. Safety, along with a safe working environment, should also be promoted at home. Overall, education on burn prevention should be intensified.
